# Association of Beta Blocker Use With Bone Mineral Density in the Framingham Osteoporosis Study: A Cross‐Sectional Study

**DOI:** 10.1002/jbm4.10388

**Published:** 2020-07-30

**Authors:** Christine W Lary, Alexandra C Hinton, Kathleen T Nevola, Theresa I Shireman, Katherine J Motyl, Karen L Houseknecht, F. Lee Lucas, Sarah Hallen, Andrew R Zullo, Sarah D Berry, Douglas P Kiel

**Affiliations:** ^1^ Center for Outcomes Research and Evaluation Maine Medical Center Research Institute Portland ME USA; ^2^ Department of Cell, Molecular, and Developmental Biology, Graduate School of Biomedical Sciences Tufts University Boston MA USA; ^3^ Center for Gerontology and Health Care Research Brown University School of Public Health Providence RI USA; ^4^ Center for Molecular Medicine Maine Medical Center Research Institute Scarborough ME USA; ^5^ Department of Biomedical Sciences, College of Osteopathic Medicine University of New England Biddeford ME USA; ^6^ Rhode Island Hospital Providence RI USA; ^7^ Department of Medicine Beth Israel Deaconess Medical CenterHarvard Medical School Boston MA USA; ^8^ Hinda and Arthur Marcus Institute for Aging ResearchHebrew SeniorLife Boston MA USA

**Keywords:** AGING, ANALYSIS/QUANTITATION OF BONE, BETA BLOCKER, DXA, EPIDEMIOLOGY, GENERAL POPULATION STUDIES

## Abstract

Some, but not all, prior observational studies have shown that beta blocker (BB) use is associated with lower fracture risk and higher bone mineral density (BMD). Rodent studies show the mechanism to involve the reduction in the effects of beta‐adrenergic signaling on bone remodeling. Because previous studies did not have detailed information on dose, duration, and beta‐1 selectivity, we examined these in a cross‐sectional analysis of the association between BB use and hip and spine BMD using DXA with the Offspring Cohort of the Framingham Heart Study. The sample size was *n* = 1520, and 397 individuals used BBs. We used propensity score modeling to balance a comprehensive set of covariates using inverse probability of treatment weighting (IPTW) to minimize bias due to treatment indication. We found significant differences in BMD between BB users and non‐users for three of four BMD measurements (femoral neck: 3.1%, 95% CI, 1.1% to 5.0%; total femur: 2.9%, 95% CI, 0.9% to 4.9%; femoral trochanter: 2.4%, 95% CI, −0.1% to 5.0%; and lumbar spine: 2.7%, 95% CI, 0.2% to 5.0%). Results were found to be similar between sexes although the magnitude of association was larger for women. Similar differences were estimated for beta‐1 selective and nonselective BBs compared with no BB use. We modeled dose in categories (no BB use, low‐dose, high‐dose) and as a continuous variable and found an increasing dose response that levels off at higher doses. Finally, associations were similar for short‐term versus long‐term (≤4 years versus >4 years) use. In summary, this large comprehensive study shows that BB use is associated with higher BMD in a dose‐related manner regardless of beta‐1 specificity and duration of use, which supports the conduct of a randomized clinical trial of BBs for achieving improvements in BMD for individuals at risk of bone loss with aging. © 2020 The Authors. *JBMR Plus* published by Wiley Periodicals LLC. on behalf of American Society for Bone and Mineral Research.

## Graphical abstract

Predicted bone mineral density (BMD) in g/cm^2^ as a function of standardized dose (defined daily dose [DDD]) in polynomial dose response models with sample weighing by inverse probability of treatment weights (IPTW).

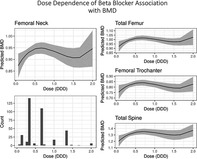

## Introduction

Osteoporosis is a skeletal disorder characterized by compromised bone strength predisposing to an increased risk of fracture, and bone strength is determined to a great extent by bone mineral density (BMD). Many observational studies have found beta blocker (BB) use to be associated with higher BMD,^(^ ^1^, ^2^, ^3^, ^4^ ^)^ although others have not observed an association.^(^ ^5^, ^6^, ^7^ ^)^ There are only a couple of randomized trials of BB use on bone outcomes such as bone turnover markers, which have shown mixed results.^(^ ^8^, ^9^ ^)^

The influence of BB treatment characteristics such as beta‐1 selectivity, dose, and length of treatment is less well studied, with inconsistent results. A randomized trial with BMD as an outcome has not yet been performed to our knowledge; such a study would be challenging given the modest effect size and the uncertain effects of beta‐1 selectivity, dose, and exposure duration. Alternatively, we conducted a cross‐sectional study of BB use on BMD of the hip and spine in a large, well‐characterized cohort of older adults who participated in the Framingham Osteoporosis Study. We included in our design two important components: (i) assessment of the effects of beta‐1 selectivity, dose, and duration of exposure; and (ii) careful control of confounders through propensity score modeling and inverse probability of treatment weighting (IPTW) with a large list of clinically‐relevant and carefully collected bone‐related characteristics, which has not typically been performed in previous studies of BB use and BMD. Thus this study was conducted to fill the gap in knowledge regarding the association of BB and BMD according to beta‐1 selectivity, dose, and length of exposure, which are important considerations for the design of future randomized trials.

## Subjects and Methods

### Framingham Osteoporosis Study

The Framingham Heart Study (FHS) was started in 1948 with the goal of studying common factors or characteristics that contribute to cardiovascular disease.^(^ ^10^ ^)^ The original cohort consisted of 5209 men and women between the ages of 30 and 62 years from the town of Framingham, Massachusetts, who had not yet developed overt symptoms of cardiovascular disease or suffered a heart attack or stroke. Since that time the study has added an offspring cohort in 1971, with the recruitment of 5124 children of members of the Framingham original cohort and the spouses of offspring, which has been examined approximately every 4 years.^(^ ^10^ ^)^ FHS Offspring participants were part of the Framingham Osteoporosis Study beginning in 1996. Osteoporosis call‐back visits have occurred for the offspring cohort at visits 7, 8, and 9. We have used data from the exam 8 call‐back visit for the current cross‐sectional study.

### Cohort definition

**Fig 1:**
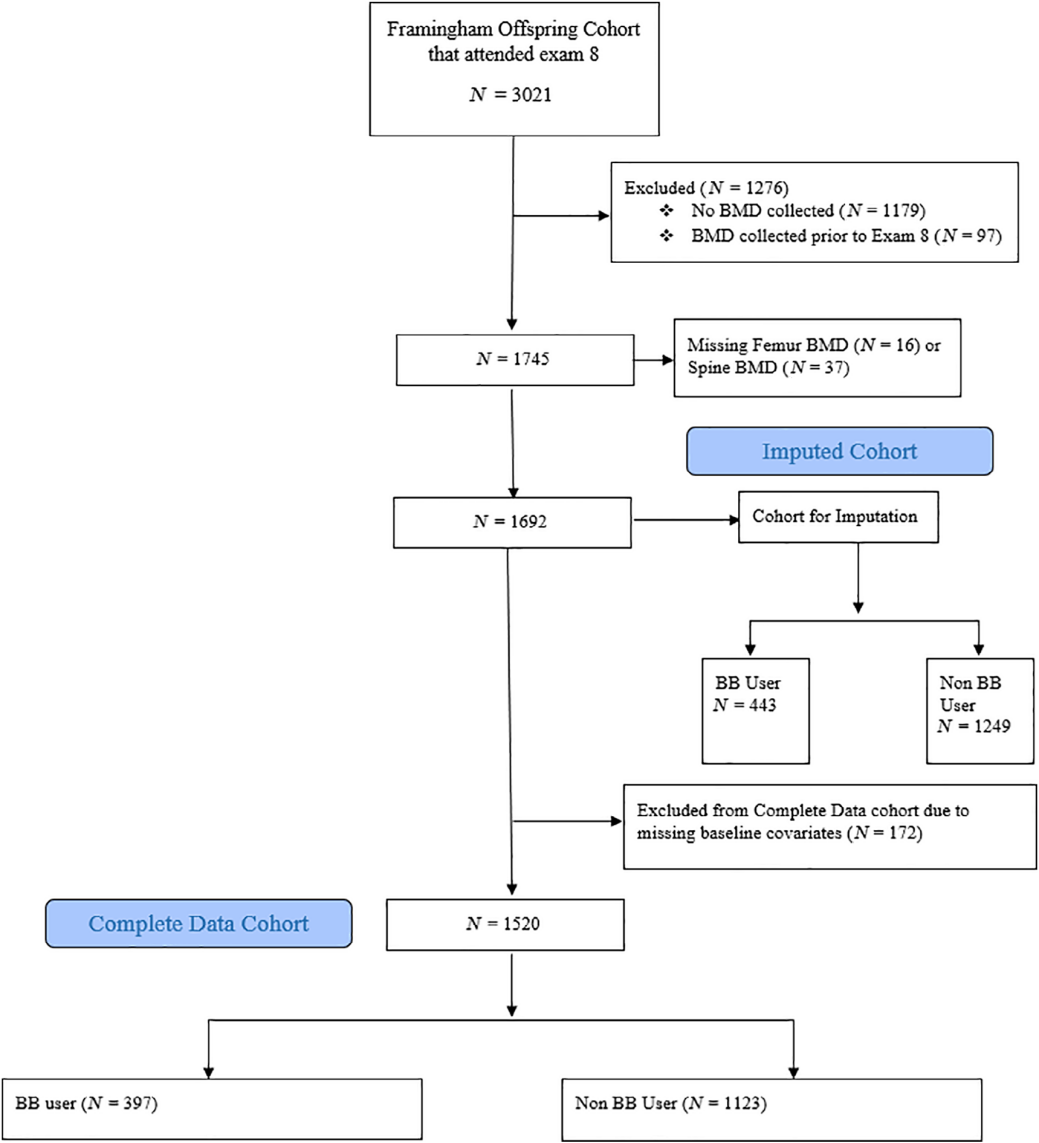
Description of study cohort.

The cohort selection is described in Fig. 1. Framingham Offspring participants who attended a “call‐back” to their regular exam 8 (between 2005 and 2008) visit, and had complete hip and spine BMD data were included (*n* = 1692). Participants with data for only hip or spine were examined in a sensitivity analysis. Cohort members were further excluded if they were missing data for baseline covariates included in the propensity score model, leaving a complete data sample of 1520 for the primary analysis. In a secondary analysis, the full cohort (or cohort for imputation) was examined using multiple imputation.

### Exposure ascertainment

BB usage was measured using a medication questionnaire in which the medication name, strength, route, and frequency (day/week/month/year) were obtained by directly viewing the medication bottle during the exam 8 (2005 to 2008) visit. We included only orally‐administered drugs, and excluded as needed (PRN) drug use. We categorized BB users as beta‐1 selective for the chemical group “beta blocking agents, selective” and as “nonselective” for the chemical groups “beta blocking agents, nonselective” or “alpha and beta blocking agents.”

We computed daily dose for each patient and for each drug by converting the strength and frequency to a daily dose. We divided this calculated daily dose by the WHO‐determined defined daily dose (DDD)^(^ ^11^ ^)^ to get a standardized dose in units of DDD for that drug. The list of BBs for this cohort along with the beta‐1 selectivity class, standardized daily dose, and number of users and dose range is shown in Table S1. Note for instance that the DDD for atenolol is 75 mg. We combined standardized doses for all BB users with valid dose data and stratified at the median, which corresponded to one‐half of a DDD (0.5 DDD), resulting in categories of low‐dose and high‐dose. Note we excluded dose data for one individual with a dose of more than two times the maximum dose. Finally, we categorized duration of BB use into: (i) the current exam only (≤4 years); (ii) use at the current and prior exam (>4 years); and (iii) or no use. In the Framingham Offspring cohort, the approximate interval between visits was 4 years. We note that individuals that had used BB at prior exams but not the current exam were classified as nonusers.

### Baseline covariates

A set of clinical covariates was included due to their documented relationship with bone outcomes, BB use, or both. These included age, weight, height, body mass index (BMI), current smoking, and self‐reported hours of moderate physical activity per week, which was chosen as a representative activity measure. In addition, from the semiquantitative Willett validated food‐frequency questionnaire,^(^ ^12^, ^13^, ^14^ ^)^ we obtained intake of caffeine, alcohol, calcium, and vitamin D. We also included current estrogen replacement for women, the use of medication for bone disease, and comorbidities, including prior cardiovascular disease or current treatment for hypertension or diabetes. Note cardiovascular disease was considered as having had a cardiovascular event prior to exam 8 not resulting in death. We included the highest educational degree, which we dichotomized as education beyond high school versus high school degree or less. Because frailty is related to fracture^(^ ^15^ ^)^ and may be associated with BMD, we also included the following covariates from the Fried frailty definition^(^ ^16^ ^)^: exhaustion (positive response to “everything I did in the past week was an effort” or “I could not get going”), gait speed (maximum time in seconds over two tries to walk a measured course), grip strength (maximum of handgrip strength of the right or left hand measured to the nearest kilogram), and unintentional weight loss (unintentional weight loss of greater than 10 pounds in the past year). The frequency of these covariates is described for our cohort in Table 1.

**Table 1:** Cohort Characteristics

Characteristic	Unadjusted (*n* = 1520)		Propensity score adjusted (*n* = 1514)^a^	
	Beta blocker use (*n* = 397)	No beta blocker use (*n* = 1123)	Beta blocker use (*n* = 390)^a^	No beta blocker use (*n* = 1124)^a^
Demographic and lifestyle factors				
Female, *n* (%)	189 (48)	638 (57)	215 (55)	613 (54)
Age (years), mean ± SD	69 ± 8	65 ± 8	66 ± 8	66 ± 9
Height (inches), mean ± SD	65.9 ± 3.7	65.8 ± 3.8	65.7 ± 3.6	65.8 ± 3.8
Weight (lbs), mean ± SD	181 ± 37	172 ± 38	174 ± 37	175 ± 39
BMI (kg/m^2^), mean ± SD	29.1 ± 5.0	27.8 ± 5.1	28.2 ± 4.9	28.2 ± 5.3
Highest degree or level of school (education beyond high school degree), *n* (%)	256 (64)	812 (72)	270 (69)	788 (70)
Current smoker, *n* (%)	28 (7.1)	94 (8.4)	27 (6.9)	89 (7.9)
Daily hours of moderate activity, mean ± SD	3.70 ± 2.17	3.74 ± 2.22	3.78 ± 2.16	3.73 ± 2.20
Beta blocker type, *n* (%)				
Beta blocker subclass				
Beta‐1 selective	344 (87)	0 (0)	335 (86)	0 (0)
Nonselective	53 (13)	0 (0)	55 (14)	0 (0)
No beta blocker use	0 (0)	1123 (100)	0 (0)	1124 (100)
Comorbid conditions and treatments, *n* (%)				
Estrogen treatment				
No estrogen	172 (43)	543 (48)	192 (49)	530 (47)
Estrogen	14 (3.5)	59 (5.3)	17 (4.3)	54 (4.8)
Male (no estrogen)	208 (52)	485 (43)	175 (45)	512 (46)
Missing	3 (0.8)	36 (3.2)	6 (1.6)	29 (2.6)
Prior cardiovascular disease^b^	146 (37)	73 (6.5)	116 (30)	88 (7.8)
Hypertension treatment^b^	352 (89)	357 (32)	340 (87)	388 (35)
Diabetes treatment	51 (13)	84 (7.5)	38 (9.8)	101 (8.9)
Taking prescription drugs for bone disease	42 (11)	159 (14)	55 (14)	149 (13)
Markers of frailty				
Gait (4 meter walk time in seconds), mean ± SD	3.89 ± 1.05	3.59 ± 0.91	3.67 ± 0.93	3.67 ± 0.98
Grip (kg, measured using JAMAR dynamometer), mean ± SD	31 ± 11	32 ± 12	31 ± 11	32 ± 12
Unintentional weight loss of 10+ lbs in the past year, *n* (%)	35 (8.8)	64 (5.7)	25 (6.3)	72 (6.4)
Exhaustion, *n* (%)	85 (21)	191 (17)	68 (17)	203 (18)
Dietary information, mean ± SD				
Daily intake of caffeine (mg)	137 ± 117	161 ± 126	151 ± 124	155 ± 124
Daily intake of alcohol (g)	11 ± 15	10 ± 15	11 ± 14	10 ± 15
Daily intake of vitamin D (mg)	493 ± 317	500 ± 336	516 ± 314	500 ± 337
Daily intake of calcium (mg)	1052 ± 545	1129 ± 587	1144 ± 586	1114 ± 581

BMI = body mass index.

^a^ Effective sample size.

^b^ Not included in propensity score model due to association with exposure.

### Outcomes

BMD of the hip and spine was measured using dual‐energy X‐ray absorptiometry (DXA) with a GE Lunar Prodigy fan‐beam densitometer (GE Healthcare, Inc., GE Healthcare, Piscataway, NJ, USA), using standard positioning recommended by the manufacturer with BMD measured in grams per centimeter squared (g/cm^2^). The right hip was scanned unless there was a history of previous fracture or hip replacement, in which case the left side was scanned. Measurements for femoral neck (FN), total femur (TF), and femoral trochanter (FT) BMD were included. Spine BMD measurements of individual lumbar vertebral levels 2, 3, and 4 and a summary average of L_2_–L_4_ were included. The coefficients of variation for FN, FT, and LS BMD were 1.7%, 2.5%, and 0.9%, respectively.^(^ ^17^, ^18^ ^)^ Not all participants had both hip and spine BMD measured at a particular exam. DXA scans occurred at a median of 58 days after the exam 8 visit.

### Statistical analyses

Our primary analysis was conducted with the complete data sample (*n* = 1520). Propensity score (PS) modeling of BB users versus nonusers was conducted using the WeightIt package in R (version 3.51; R Foundation for Statistical Computing, Vienna, Austria; https://www.r-project.org/) with a minimum standardized difference of 0.1 required between treatment groups in the weighted sample. Propensity score models were built using main effects of all covariates except treatment for hypertension and prior cardiovascular disease (CVD), which were excluded due to their high correlation with the exposure and lack of association with the outcomes. They were included in an outcomes model for those treated for hypertension, which was performed as a sensitivity analysis. The linear terms for continuous covariates were included in the main analysis, with spline terms for continuous covariates with the strongest additive effects including age, BMI, gait, caffeine, and calcium additionally included in another sensitivity analysis. The spline was computed using the b spline basis matrix using the function bs in the splines package. Inverse probability of treatment weighting (IPTW) was used to incorporate the PS weight in each outcome model. Balance in covariates in the weighted sample was assessed using the bal.tab function in R.

Multiple imputation for missing baseline covariates in the imputed cohort was conducted using the mice package in R with 10 imputed samples and propensity score balancing performed within each imputed data set. To perform the outcomes analysis we used the method recommended by Mitra and Reiter^(^ ^19^ ^)^ in 2016, in which we averaged the PS scores for each individual across imputed data sets, computed the resulting weight per individual, and used this weight in each outcomes analysis.

Outcomes analysis for each of the four outcomes was performed using the svyglm package in R using the IPTW weights from the PS model. The primary outcomes model was a linear model with each continuous BMD outcome as a function of BB use in which balance across all covariates was achieved (see Table 1). Additional models were constructed as sensitivity analyses for the primary outcomes model as in the Subject and Methods/Statistical analyses section and include a model including those with missing baseline covariates using multiple imputation, a model for only those treated for hypertension, a model for those with complete hip *or* spine outcomes data, and a model where spline terms were included in the PS model. Additional covariates were included in the additional models if balance was not achieved for those covariates.

### Analysis by sex or by BB characteristics

Sex‐stratified models were generated for the original exposure (BB use), and new exposures were created to examine specific BB characteristics. These included a beta‐1 selectivity model (categories of beta‐1 selective, nonselective, or nonuser), a dose model (categories of greater than the median standardized daily dose, less than the median, or nonuser), and a length of use model (current use, current and prior use, or no use). For the sex‐stratified model, we refit the PS models within sex and performed the outcomes analyses in each sex. For the new exposure analyses, we refit the PS model using WeightIt for a multinomial treatment with three categories using pairwise logistic regression rather than multinomial logistic regression in which we were unable to achieve good covariate balance. We then fit each of our outcomes models with these new exposures and corresponding weights. For dose, we additionally modeled outcomes as a function of continuous dose after computing IPTW weights from a continuous dose model for BB users, including linear, quadratic, and cubic terms for dose. Note we excluded one outlier from this continuous dose model with standardized dose of greater than two.

## Results

The construction of the cohort is described in Fig. 1. There were 1692 individuals with exam 8 data and complete hip and spine BMD. When further requiring complete baseline data, the cohort size dropped to *n* = 1520 and is described in Table 1 (the complete data cohort). The complete data sample had an average age of 66 ± 9 years (mean ± SD) and was 54% female. BBs were used by 397 (26%). BB users were older, more likely to be male, had higher BMI, and had a greater rate of diabetes, prior cardiovascular disease (CVD), and hypertension. The current smoking rate was slightly lower in BB users but they had higher levels of unintentional weight loss and exhaustion reported. BB users had slightly lower rates of estrogen use and use of medication for bone disease. To account for these systematic differences in covariates between exposure groups, we used propensity score balancing to adjust for these confounders. The covariate balance in the weighted samples is much improved as shown in the second two columns in Table 1. All covariates had standardized differences less than 0.1 between groups in the weighted cohort in the primary analysis (data not shown).

### Differences in BMD with BB use

The results of the primary outcomes analysis are shown in Table 2 as the estimated difference between BB users and nonusers, including the predicted means of each group. BB use was associated with significantly higher BMD across three of four BMD measurements, with femoral trochanter not showing significance. The percentage difference was 3.1% (95% CI, 1.1% to 5.0%) for femoral neck, 2.9% (95% CI, 0.9% to 4.9%) for total femur, 2.4% (95% CI, −0.1% to 5.0%) for femoral trochanter, and 2.7% (95% CI, 0.2% to 5.0%) for lumbar spine. The estimated differences stratified by sex were similar though slightly higher in females, although no significant interaction with sex was found when adding sex to the main model (data not shown). Significant differences in BMD were seen in women at the femoral neck and total femur, whereas no skeletal sites showed significant differences in men. To illustrate the sex‐specific differences, the difference in femoral neck BMD for female BB users compared with nonusers was 3.7%, which was only 1.5% for male BB users compared with nonusers.

**Table 2:** Predicted Mean and Estimated Percentage Difference Between Beta Blocker Users Compared With Nonusers and *p* for Multiple Measures of BMD, Overall and Stratified by Sex, After IPTW Weighting

Population	Estimated mean BMD (g/cm^2^)		Percent difference from nonusers (95% CI)	*p*
	Nonusers	Users		
Femoral neck				
Overall	0.903	0.931	3.10 (1.11 to 4.98)	0.002
Female	0.857	0.890	3.73 (1.05 to 6.53)	0.006
Male	0.964	0.978	1.45 (−1.45 to 4.25)	0.337
Femoral trochanter				
Overall	0.783	0.802	2.43 (−0.13 to 4.98)	0.062
Female	0.703	0.720	2.42 (−0.71 to 5.55)	0.122
Male	0.884	0.894	1.13 (−2.15 to 4.30)	0.507
Total femur				
Overall	0.959	0.987	2.92 (0.94 to 4.90)	0.004
Female	0.898	0.925	3.01 (0.33 to 5.57)	0.028
Male	1.039	1.055	1.54 (−1.15 to 4.33)	0.264
Total spine				
Overall	1.246	1.278	2.65 (0.24 to 4.98)	0.032
Female	1.154	1.185	2.77 (−0.26 to 5.81)	0.077
Male	1.362	1.390	2.06 (−1.47 to 5.58)	0.257

BMD = bone mineral density; IPTW = inverse probability of treatment weights.

In order to assess the dependence of our conclusions on modeling choices and assumptions, we performed a number of sensitivity analyses as described in the Subjects and Methods section. To account for potential bias due to the lack of inclusion of patients with missing baseline covariates, we used multiple imputation of these covariate values and performed an outcomes analysis with the full cohort (Table S2). The results of this analysis show slightly smaller estimated differences, but the overall conclusions remained the same with the same three of four traits showing significance. We showed that our results were similar in a cohort of individuals treated for hypertension, and in cohorts with only complete hip or complete spine data (and with complete baseline covariates). Finally, we sought to examine the sensitivity of our results to choice of propensity score model, which was relatively simple. The results of a more complicated PS model with spline terms are shown in Table S2 and show very little difference with the main analysis results.

### Beta‐1 selectivity, dose, and duration of use

**Fig 2:**
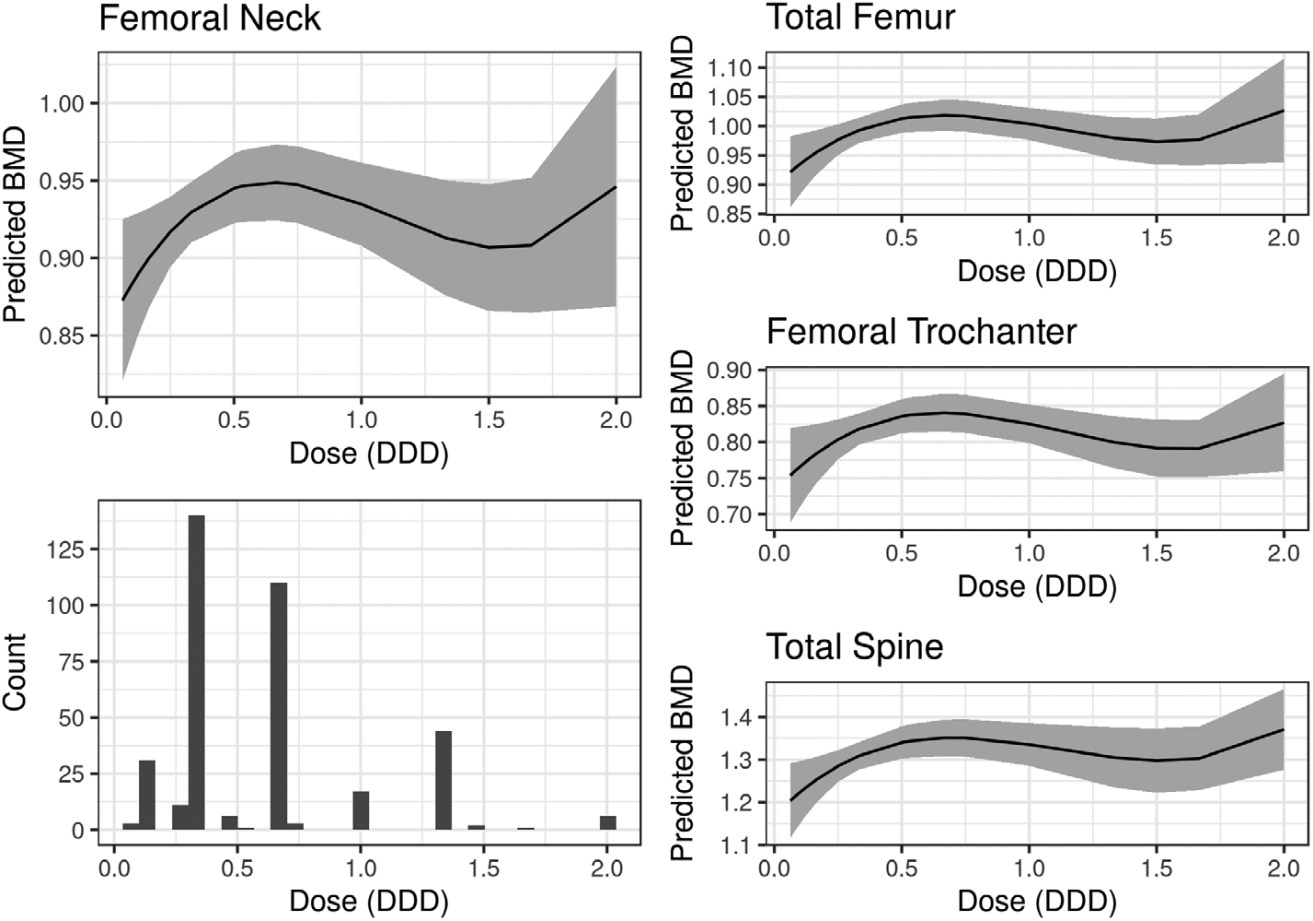
Predicted BMD in g/cm^2^ as a function of dose standardized to the DDD for each drug for femoral neck, total femur, femoral trochanter, and total spine, and counts of individuals at each dose. Samples are weighted by IPTW from a continuous dose model, and dose response was measured using linear, quadratic, and cubic terms (see parameter estimates in Table S3). One outlier sample with standardized dose >2 was excluded from the dose model and plots. BMD = bone mineral density; DDD = defined daily dose; IPTW = inverse probability of treatment weights.

Next, we examined the association between beta‐1 selectivity, dose, and length of use on the association of BB and BMD. The estimated differences in BMD for beta‐1 selective and nonselective BB users compared with nonusers (Table 3 **)** were similar with overlapping confidence intervals. The beta‐1 selective estimated difference was more precise with a lower *p* value due to the large prevalence in this cohort (87%, see Table 1), but no significant differences were seen between beta‐1 selective and nonselective BB users. The direct estimates of percentage difference between beta‐1 selective and nonselective users is −1.2% (95% CI, −4.9% to 2.6%), −1.5% (95% CI, −5.2% to 2.3%), −1.5% (95% CI, −5.9% to 2.7%), and −0.5% (95% CI, −6.3% to 5.2%), for femoral neck, total femur, femoral trochanter, and total spine, respectively, and all were found to be nonsignificant.We then examined the effect of dose of BB first by modeling the categories of no use and low‐dose and high‐dose categories. As shown in Table 4, although the confidence intervals were overlapping for the low‐dose and high‐dose categories, the estimated differences were higher for the high‐dose categories for all four BMD measurements. For instance, for femoral neck BMD, the low‐dose group had a percentage difference of 3.1% (95% CI, 0.6% to 5.7%) whereas the high‐dose group had an estimated difference of 4.2% (95% CI, 1.4% to 7.0%). To explore in more detail the dependence of BMD on dose, we fit polynomial models using continuous dose (see Subjects and Methods) which showed increasing BMD from low to intermediate doses but then leveling off or decreasing slightly at higher doses (see Fig. 2, Table S3).

**Table 3:** Predicted Mean and Estimated Percentage Difference Between Beta‐1 Selective and Nonselective Users Compared With Nonusers and *p* for Multiple Measures of BMD After IPTW Weighting

Parameter	*n*	Estimated mean BMD	Percent difference from nonusers (95% CI)	*p* ^a^
Femoral neck				
No beta blocker	1123	0.903	0 (Referent)	
Nonselective	53	0.944	4.43 (1 to 7.86)	0.011
Beta‐1 selective	344	0.933	3.32 (1.44 to 5.09)	<0.001
Total femur				
No beta blocker	1123	0.960	0 (Referent)	
Nonselective	53	1.005	4.79 (1.25 to 8.23)	0.008
Beta‐1 selective	344	0.991	3.23 (1.46 to 5.1)	0.001
Femoral trochanter				
No beta blocker	1123	0.783	0 (Referent)	
Nonselective	53	0.818	4.47 (0.51 to 8.43)	0.025
Beta‐1 selective	344	0.806	2.94 (0.64 to 5.24)	0.012
Total spine				
No beta blocker	1123	1.246	0 (Referent)	
Nonselective	53	1.286	3.21 (−2.33 to 8.75)	0.262
Beta‐1 selective	344	1.280	2.65 (0.4 to 4.98)	0.023

BMD = bone mineral density; IPTW = inverse probability of treatment weights.

^a^ Value of *p* is for the comparison with the “no beta blocker use” group.

**Table 4:** Difference Between Low‐Dose and High‐Dose BB Users Compared With Nonusers and BMD After IPTW Weighting

Parameter	*n*	Estimated mean BMD	Percent difference from nonusers (95% CI)	*p* ^a^
Femoral neck				
No beta blocker use	1123	0.903	0 (Referent)	
Low dose (≤0.5 DDD)	191	0.931	3.10 (0.55 to 5.65)	0.017
High dose (>0.5 DDD)	185	0.941	4.21 (1.44 to 6.98)	0.003
Total femur				
No beta blocker use	1123	0.959	0 (Referent)	
Low dose (≤0.5 DDD)	191	0.988	3.02 (0.31 to 5.74)	0.029
High dose (>0.5 DDD)	185	1.001	4.48 (1.77 to 7.09)	0.001
Femoral trochanter				
No beta blocker use	1123	0.782	0 (Referent)	
Low dose (≤0.5 DDD)	191	0.802	2.56 (−1.02 to 6.01)	0.157
High dose (>0.5 DDD)	185	0.815	4.22 (1.02 to 7.29)	0.008
Total spine				
No beta blocker use	1123	1.246	0 (Referent)	
Low dose (≤0.5 DDD)	191	1.278	2.65 (−0.56 to 5.78)	0.106
High dose (>0.5 DDD)	185	1.284	3.05 (−0.40 to 6.58)	0.082

Predicted mean and estimated percentage difference between low‐dose (≤0.5 DDD) and high‐dose (>0.5 DDD) users compared with nonusers, and *p* value for multiple measures of BMD (in g/cm^2^) after weighting by IPTW.

BMD = bone mineral density; DDD = defined daily dose; IPTW = inverse probability of treatment weights.

^a^ Value of *p* is for the comparison with the “no beta blocker use” group.

Finally, we examined how the duration of exposure influenced the association of BB use and BMD. We categorized exposure duration of BB use as current use (≤4 years), current and prior use (>4 years), or no use, and observed similar estimated differences between BB users of different exposure durations with nonusers with overlapping 95% CIs (Table S4).

## Discussion

The purpose of this study was to assess the association between BB use and BMD of the hip and spine and to measure how this association is modified by beta‐1 selectivity, dose, and duration of use, using rigorous statistical methods in a large cross‐sectional study of older adults. Using propensity score‐weighted models in the Framingham Osteoporosis Study, we showed significantly higher BMD for BB users compared with nonusers for three of the four bone sites (femoral neck, total femur, and lumbar spine). The associations were slightly greater for women compared with men. We showed similar differences for beta‐1 selective BBs and nonselective BBs as compared with no use. In terms of dose, our results showed increasing association from low to intermediate doses with leveling off or decreasing of the association at the highest doses. Finally, we found no association between the duration of BB use and BMD differences.

We found that on average women who used BB had a 3.7% increase in femoral neck BMD as compared with nonusers. The magnitude of this difference is roughly six times the average annual loss of femoral neck BMD of 0.6% in older men and women (average age 75 years), although rates vary by age.^(^ ^20^ ^)^ Thus if the association is causal, any use of a BB as ascertained in our study compared to nonuse is equivalent to reversing the typical decreases in BMD due to aging occurring over 6 years. It should be noted that the rate of bone loss in women may be higher closer to the transition to menopause, and thus the estimated number of years of bone loss for younger women may be lower. It should also be noted that future studies should investigate the effect of age on the association of BB with BMD given the variation of bone loss with age.

Our results largely confirm results from the prior literature, with some important caveats. In terms of our primary outcomes analysis, our finding of a significant positive association of BB and BMD is consistent with many prior studies^(^ ^1^, ^2^, ^3^, ^4^ ^)^ although not all.^(^ ^5^, ^6^, ^7^ ^)^ Our effect estimates are similar to those from previous positive studies.^(^ ^1^, ^2^, ^3^, ^4^ ^)^ For instance, our estimate and the range from previous studies are 3.1% (range, 2.5% to 4.5%) for femoral neck, 2.9% (range, 2.5% to 6.3%) for total femur, and 2.6% (range, 3.2% to 8.4%) for lumbar spine. The positive studies were either cross‐sectional^(^ ^2^, ^3^ ^)^ or case control,^(^ ^1^, ^4^ ^)^ with sample sizes ranging from 150 to 3500 and with BB use in 12% to 33% of the sample. For the negative studies,^(^ ^5^, ^6^, ^7^ ^)^ one was cross‐sectional^(^ ^6^ ^)^ with a very small number BB users, and the other two were longitudinal, one with a small number of BB users,^(^ ^7^ ^)^ and another with a larger number.^(^ ^5^ ^)^ Thus the results of our cross‐sectional study are in line with previous cross‐sectional and case control studies except for one which had an extremely small number of BB users. Note that in addition to differences in sample size, exposure rate, and study design already noted, there were also differences in study population (women‐only versus sex combined and older versus elderly adults), methods for accounting for confounders (regression adjustment versus propensity score weighting), and variability in type or strength of BBs (many different types and doses reported). For the negative studies, there was either a small sample size of BB users^(^ ^6^, ^7^ ^)^ or BB use was no longer found to be significant after correction for confounders.^(^ ^5^ ^)^ However, this last study used simple regression methods for confounder adjustment, which can be problematic due to model misspecification. Propensity score adjustment is thought to be the most effective way of handling confounding due to treatment indication.^(^ ^21^, ^22^ ^)^

Our study extended previous results by determining that the association between BB use and bone density was not based on beta‐1 selectivity of the BB, although our sample size of nonselective users was limited. Most studies that have noted selectivity have found beta‐1–selective BBs to show stronger positive associations,^(^ ^3^, ^4^, ^7^ ^)^ although it should be noted that no relationship with BB effect and beta‐1 selectivity was found in some studies.^(^ ^9^, ^13^ ^)^ It should also be noted that the vast majority of BB users in the current study were beta‐1–selective users (87%), and thus the strong positive associations that we measured are largely due to these users. Given the number of beta‐1–selective and nonselective users in our study, we were powered to detect a 5.3% increase between the two classes for femoral neck BMD, which is larger than the total effect size observed of 2.8%, thus we were underpowered for this contrast. This is largely driven by the small number of nonselective users (53) in our sample. Another limitation is that because this was not a new user design, users in each medication class could have had prior exposure to the opposite class. Of the 53 nonselective users, 10 were on a beta‐1 selective BB at the prior exam, thus effects from prior use may have served to reduce the estimate of the difference between these two classes, although this difference would be expected to be small.

Although our results suggesting BB use may be protective against bone loss are encouraging, existing literature suggests that these drugs may be associated with an increased risk of falls.^(^ ^23^ ^)^ The results of our study can be used to inform the design of a randomized clinical trial of BB use to prevent BMD decline with aging, with careful monitoring of falls and fractures. For instance, the differences in femoral neck BMD observed between BB users and nonusers (3.1% overall and 3.7% for women) could be used as effect estimates for the power calculations of these studies, and a study first in women may be prudent given the larger association seen in women. In addition, our results indicate larger associations at intermediate to high doses, so we would recommend a dosage of at least two‐thirds of a DDD, corresponding to a daily dose of 50 mg of atenolol or 100 mg metoprolol, for example. Finally, our results were not informative regarding duration of use, so previous estimates of at least 3 months of use are still applicable.^(^ ^24^ ^)^ Finally, given the previously noted risk of falls^(^ ^23^ ^)^ and other adverse outcomes associated with BB use in older adults (>65 years),^(^ ^25^ ^)^ it would be recommended to conduct a trial in adults younger than 65.

Our study has several strengths including the large sample size of BB users and nonusers with a collection of detailed bone BMD measurements via DXA and rich clinical covariates, availability of medication details such as type of BB drug, dose, and duration of use, and comprehensive statistical adjustment for confounders using propensity score modeling. Our study also has limitations that should be noted. These include the largely cross‐sectional nature of the design with BMD collected shortly after ascertainment of the medication status, although stratified analyses for longer‐term use of BBs showed similar effect sizes as recent use. Other limitations include the lack of time resolution with regard to when the medication was started, the lack of information on medication adherence, and the limited sample size for some of the more detailed subanalyses, including the sex‐specific analyses and the analysis of nonselective beta blocker use, which had a small number of users and contained a mixture of beta and alpha targeted agents. It should be noted that larger studies or male‐specific studies are needed to confirm a significant BB association in men. Because the cohort was of European ancestry, our findings cannot necessarily be generalized to individuals of non‐European ancestry. We note an important possibility of residual confounding due to unmeasured confounders, as propensity score modeling can only balance the treatment groups on measured confounders, thus the presence of unmeasured confounders of large effect could potentially change the results.

In summary, our study supports a positive association between BB use and higher BMD of the hip and spine. The association was dose‐related, but not related to the duration of use or beta‐1 selectivity. The implications of this study are that BB treatment, or other potential nonpharmacologic treatments targeting beta‐adrenergic signaling in bone, are a promising approach to preventing BMD loss that occurs with aging and should be investigated in a randomized control trial.

## Disclosures

DPK has received royalty payments from Wolters Kluwer for authoring a chapter in UpToDate on Falls; he has received grant funding through a grant to his Institute by the Dairy Council, Amgen, Inc, and Radius Health, and he serves on the scientific advisory board for Solarea Bio. SDB has received royalty payments from Wolters Kluwer. The other authors have no disclosures or conficts of interest to report.

## Peer Review

The peer review history for this article is available at https://publons.com/publon/10.1002/jbm4.10388.

## Supporting information


**Table S1**. Characteristics of beta blocker use in study cohort including drug name, selectivity class, defined daily dose, number of users found in cohort, and median and range of dose found in study cohort.
**Table S2**. Results of sensitivity analyses.
**Table S3**. Association between the dose of beta blocker use and bone mineral density estimated from a continuous dose model with linear, quadratic, and cubic terms, with samples weighted by IPTW (Inverse Probability of Treatment Weights) from a continuous dose model.
**Table S4**. Predicted mean and estimated percentage difference between current BB users (<4 years) and long‐term BB users (≥4 years) compared with non‐users and *p*‐value for multiple measures of bone mineral density (BMD) in g/cm^2^ after weighting by IPTW (Inverse Probability of Treatment Weights).
