# Supplementary material for: Association of Beta Blocker Use With Bone Mineral Density in the Framingham Osteoporosis Study: A Cross‐Sectional Study
Source: JBMR Plus. 2020 Jul 30;4(9):e10388. doi: 10.1002/jbm4.10388 (PMC7507481; doi:10.1002/jbm4.10388)
Supplement: Supplementary file 1 — Table S1. Characteristics of beta blocker use in study cohort including drug name, selectivity class, defined daily dose, number of users found in cohort, and median and range of dose found in study cohort. Table S2. Results of sensitivity analyses. Table S3. Association between the dose of beta blocker use and bone mineral density estimated from a continuous dose model with linear, quadratic, and cubic terms, with samples weighted by IPTW (Inverse Probability of Treatment Weights) from a continuous dose model. Table S4. Predicted mean and estimated percentage difference between current BB users (<4 years) and long‐term BB users (≥4 years) compared with non‐users and p‐value for multiple measures of bone mineral density (BMD) in g/cm2 after weighting by IPTW (Inverse Probability of Treatment Weights). [file JBM4-4-e10388-s001.docx]

**Table S1**. Characteristics of beta blocker use in study cohort including drug name, selectivity class, defined daily dose, number of users found in cohort, and median and range of dose found in study cohort.

| **Drug Name** | **Defined Daily Dose (mg)** | **N** | **Median Dose (Min, Max) (mg)** |
| --- | --- | --- | --- |
| **Beta-1 Selective** | | | |
| Acebutolol | 400.0 | 1 | 400 (400, 400) |
| Atenolol | 75.0 | 191 | 50 (12, 200) |
| Bisoprolol | 10.0 | 6 | 4 (2, 5) |
| Metoprolol | 150.0 | 146 | 50 (12, 300) |
| **Non-Selective** | | | |
| Carvedilol | 37.5 | 11 | 25 (12, 50) |
| Labetalol | 600.0 | 6 | 500 (200, 800) |
| Nadolol | 160.0 | 10 | 40 (20, 320) |
| Pindolol | 15.0 | 2 | 15 (10, 20) |
| Propranolol | 160.0 | 16 | 40 (10, 160) |
| Sotalol | 160.0 | 5 | 160 (80, 160) |
| Timolol | 20.0 | 3 | 10 (10, 10) |

**Table S2**. Results of sensitivity analyses.

| **Model** | **Bone Mineral Density (g/cm^2^)** | | | |
| --- | --- | --- | --- | --- |
|  | **Femoral Neck** | **Total Femur** | **Femoral Trochanter** | **Total Spine** |
| Main Analysis | N=1520  β=0.0276 (0.0104, 0.0448)  p=0.002 | N=1520  β=0.0282 (0.0089, 0.0474)  p=0.004 | N=1520  β=0.0191 (-0.0009, 0.0391)  p=0.062 | N=1520  β=0.0325 (0.0029, 0.0621)  p=0.032 |
| Imputation | N=1692  β=0.0238 (0.0078, 0.0398)  p=0.004 | N=1692  β=0.0254 (0.0075, 0.0433)  p=0.006 | N=1692  β=0.0167 (-0.0019, 0.0354)  p=0.079 | N=1692  β=0.0261 (-0.0016, 0.0538)  p=0.064 |
| Hypertension Subset | N=709  β=0.0334 (0.0116, 0.0553)  p=0.003 | N=709  β=0.0302 (0.0051, 0.0553)  p=0.019 | N=709  β=0.0227 (-0.0028, 0.0482)  p=0.081 | N=709  β=0.0289 (-0.0095, 0.0673)  p=0.14 |
| Complete Outcomes | N=1550  β=0.0266 (0.0097, 0.0436)  p=0.002 | N=1550  β=0.0276 (0.0087, 0.0464)  p=0.004 | N=1550  β=0.0190 (-0.0006, 0.0385)  p=0.057 | N=1532  β=0.0332 (0.0036, 0.0629)  p=0.028 |
| Propensity Score with Spline Terms | N=1520  β=0.0231 (0.0053, 0.0410)  p=0.011 | N=1520  β=0.0250 (0.0047, 0.0453)  p=0.016 | N=1520  β=0.0165 (-0.0045, 0.0375)  p=0.12 | N=1520  β=0.0316 (0.0009, 0.0624)  p=0.044 |

**Table S3**. Association between the dose of beta blocker use and bone mineral density estimated from a continuous dose model with linear, quadradic, and cubic terms, with samples weighted by IPTW (Inverse Probability of Treatment Weights) from a continuous dose model.

| **Characteristic**, N = 375 | **Terms** | **Beta** | **95% CI***^1^* | **p-value** |
| --- | --- | --- | --- | --- |
| Femoral Neck | Linear | 0.344 | 0.043, 0.645 | 0.026 |
|  | Quadratic | -0.375 | -0.722, -0.028 | 0.035 |
|  | Cubic | 0.113 | 0.001, 0.226 | 0.049 |
| Total Femur | Linear | 0.428 | 0.087, 0.768 | 0.014 |
|  | Quadratic | -0.460 | -0.844, -0.075 | 0.020 |
|  | Cubic | 0.139 | 0.015, 0.263 | 0.029 |
| Femoral Trochanter | Linear | 0.386 | 0.030, 0.741 | 0.034 |
|  | Quadratic | -0.414 | -0.809, -0.020 | 0.040 |
|  | Cubic | 0.123 | -0.002, 0.247 | 0.054 |
| Total Spine | Linear | 0.618 | 0.110, 1.13 | 0.018 |
|  | Quadratic | -0.642 | -1.23, -0.058 | 0.032 |
|  | Cubic | 0.192 | 0.006, 0.377 | 0.043 |
| *^1^*CI = Confidence Interval | | | | |

**Table S4**. Predicted mean and estimated percentage difference between current BB users (<4 years) and long-term BB users (≥4 years) compared with non-users and *p*-value for multiple measures of bone mineral density (BMD) in g/cm^2^ after weighting by IPTW (Inverse Probability of Treatment Weights).

**p value is for the comparison with the No Beta Blocker group.*

|  | **N** | **Estimated Mean BMD** | **Percent Difference from Non-Users (95% CI)** | **p* value** |
| --- | --- | --- | --- | --- |
| **Femoral Neck** | | | | |
| No Beta Blocker Use | 1123 | 0.903 | 0 (Referent) |  |
| <4 years | 201 | 0.944 | 4.65 (2.1, 7.09) | 0.000 |
| ≥4 years | 185 | 0.926 | 2.55 (-0.33, 5.32) | 0.079 |
| **Total Femur** | | | | |
| No Beta Blocker Use | 1123 | 0.959 | 0 (Referent) |  |
| <4 years | 201 | 0.993 | 3.55 (1.04, 6.05) | 0.006 |
| ≥4 years | 185 | 0.990 | 3.23 (0.42, 6.05) | 0.027 |
| **Femoral Trochanter** | | | | |
| No Beta Blocker Use | 1123 | 0.783 | 0 (Referent) |  |
| <4 years | 201 | 0.807 | 3.07 (-0.26, 6.39) | 0.067 |
| ≥4 years | 185 | 0.803 | 2.68 (-0.89, 6.26) | 0.148 |
| **Total Spine** | | | | |
| No Beta Blocker Use | 1123 | 1.246 | 0 (Referent) |  |
| <4 years | 201 | 1.291 | 3.61 (0.64, 6.58) | 0.016 |
| ≥4 years | 185 | 1.280 | 2.73 (-0.56, 6.1) | 0.105 |
